# Sociodemographic correlates of cognitive performance in healthy children and adolescents

**DOI:** 10.3389/fpsyg.2025.1656310

**Published:** 2025-11-20

**Authors:** Elisabeth Mai, Christof Meigen, Ricarda Schmidt, Juliane Ludwig, Tanja Poulain, Wieland Kiess

**Affiliations:** 1 LIFE—Leipzig Research Center for Civilization Diseases, Leipzig University, Leipzig, Germany; 2 German Center for Child and Adolescent Health (DZKJ), Partner Site Leipzig/Dresden, Leipzig, Germany; 3 Behavioral Medicine Research Unit, Integrated Research and Treatment Center Adiposity Diseases, Department of Psychosomatic Medicine and Psychotherapy, Leipzig University Medical Center, Leipzig, Germany; 4 Department of Women and Children’s Health, Hospital for Children and Adolescents and Center for Pediatric Research (CPL), Leipzig University, Leipzig, Germany

**Keywords:** adolescents, children, cognitive tests, Continuous Performance Test, healthy cohort, Mental Rotation Test, Tetris, Trail Making Test

## Abstract

**Background:**

The maturation of cognitive abilities, a key aspect of childhood development, is associated with numerous outcomes later in life. Socioeconomic variables have been shown to influence this developmental trajectory. Given the growing global socioeconomic inequality, it is essential to account for socioeconomic factors when conducting research on cognitive development. While previous studies often focused on single cognitive domains and small cohorts or age groups, this study aimed to assess the association between sex, maternal education, and cognitive performance using three standard cognitive tests in a large cohort of healthy children and adolescents and to explore Tetris as a game-based cognitive test.

**Methods:**

Cognitive performance was examined in 9- to 19-year-olds using four tests (Trail Making Test, Mental Rotation Test, Continuous Performance Test, Tetris). Data were collected between October 2019 and December 2023 as part of the LIFE Child study, conducted in Leipzig (Germany), involving 770 participants for Tetris, Trail Making, and Mental Rotation, and 888 participants for the Continuous Performance Test. Multiple regression models, adjusted for age, considered sex and maternal education as independent variables.

**Results:**

Younger boys showed lower attention and inhibition control than girls, but improved during puberty, ultimately outperforming girls in the Continuous Performance Test. In Tetris, boys cleared more lines but made more rotation and movement faults than same-aged girls. No sex differences were observed in the Trail Making and Mental Rotation Test. Children of mothers with higher education made fewer errors in the Mental Rotation Test and were more attentive and less impulsive in the Continuous Performance Test than children of mothers with lower education. Tetris performance was significantly associated with the Mental Rotation and Trail Making Test.

**Conclusion:**

Cognitive performance patterns varied by sex and maternal education, highlighting the importance of distinguishing typical developmental variation from cognitive delay to guide individual support. Lower maternal education emerged as a potential risk factor for poorer cognitive performance, relevant for targeted interventions. We recommend further investigating Tetris as a game-based cognitive test.

## 1 Introduction

The word “cognition” is derived from the Latin word “cognoscere,” which means to realize or to experience (Bayne et al., 2019). In general, cognitive functions are defined as brain-based mental processes that encompass perceiving, acquiring, storing, transforming, and processing information (Anderson, 1991; Groome, 2013). Consequently, cognitive abilities build the foundation for performing tasks of any complexity, shaping the daily life and educational paths of children and adolescents. More specifically, cognitive abilities can be divided into lower-order and higher-order cognitive abilities. Higher-order cognitive abilities involve more complex functions, for instance, strategy development, decision making, and reasoning. Lower-order cognitive functions include basic mental processes such as attention, memory, and perception (Amudha et al., 2016). Naturally, lower-order cognitive abilities and higher-order functions operate in constant interaction in everyday tasks or in the resolution of more complex problems (Werning et al., 2013). In this study, we aimed to cover lower-order and higher-order cognitive functions, which were reported as important predictors of developmental outcomes in later life. Therefore, three established cognitive tests were applied: The Continuous Performance Test, assessing attention, impulsivity, and inhibition control (Shaked et al., 2020); the Trail Making Test, measuring cognitive flexibility, processing speed, and inhibition (Arango-Lasprilla et al., 2017); and the Mental Rotation Test, evaluating cognitive flexibility and visual–spatial working memory (Assari, 2020).

Overall, the maturation of cognitive functions plays an important role in child development and is essential for long-term outcomes, including well-being and mental health (Tuerk et al., 2023). Attention control develops rapidly in early childhood from around 12 months of age, with substantial improvement by middle childhood up to 6 years of age, which aligns with neurophysiological changes in the prefrontal cortex (Anderson, 2002; Rueda and Posner, 2013). Better attention in childhood was linked to better self-rated health and fewer illnesses at 35 in a cohort of 569 participants (Kubzansky et al., 2009). Furthermore, problems in sustained attention in children, aged 4 to 16, were associated with socioeconomic disadvantages in 22- to 35-year-old adults (Galéra et al., 2012). In a large U.S. population-based sample of 2,716 participants, attention problems at age 9 also indicated higher risks of cigarette smoking and delinquent behavior at age 15 (Casseus et al., 2025). In contrast, cognitive flexibility and processing speed showed substantial improvements during primary school and reached relative maturity by age 12 (Nettelbeck and Wilson, 1985; Anderson, 2002). Cognitive flexibility continued to develop through adolescence, paralleling the maturation of prefrontal neural networks (Buttelmann and Karbach, 2017), and was linked to various important life outcomes, including academic achievement in reading and math among school-aged children (Yeniad et al., 2013; Buttelmann and Karbach, 2017). Additionally, improvements in fluid intelligence in a sample of 7- to 19-year-olds (*n* = 214) were mediated by developmental changes in processing speed. Research indicated that the development of inhibitory control emerged within the first year of life and improved rapidly in preschool age, with the rate of improvement slowing around age 6 (Geeraerts et al., 2021). Better inhibition control at age 7 was linked to better psychosocial, cognitive, and weight outcomes in adolescence in a study with 192 participants (Anzman-Frasca et al., 2015). Regarding the development of spatial abilities, significant changes occurred from birth to school age, particularly as the children’s opportunities to move and interact with the environment increased (Okamoto et al., 2015). This developmental process was shaped by biological, psychological, sociological, and cultural factors (Okamoto et al., 2015). Visual–spatial abilities were associated with later mathematics skills and overall success in science, technology, and engineering fields (Verdine et al., 2017; Cui and Guo, 2022). These findings, combined with the knowledge that cognitive abilities can be trained during childhood (Hsu and Jaeggi, 2014; Karbach, 2015), highlighted the potential for targeted interventions to support child development, health, and well-being (Anzman-Frasca et al., 2015).

It is important to note that cognitive abilities should not be viewed as innate and fixed modules. According to the neuroconstructivist framework by Karmiloff-Smith (1994), cognitive abilities development is shaped by interactions with the environment, such as parental education or different socio-cultural experiences related to the child’s sex. In the present study, we aimed to provide results from a large cohort of healthy children and adolescents to contribute to the ongoing discussion about cognitive abilities in childhood and their associations with sex and sociodemographic factors to understand the variability in cognitive development.

Previous research on sex differences in cognitive tests, as a widely discussed topic, revealed mixed results. Some studies showed that girls were more attentive between the ages of 9 and 17 (*n* = 816) (Conners et al., 2003), while boys excelled in a paper-based visual–spatial task at the age of 10–20 (*n* = 861) (Quaiser-Pohl et al., 2006) and tended to act more impulsively (Conners et al., 2003). Conversely, other studies did not find significant sex differences in computerized visual–spatial tasks in 14 and 15-year-old adolescents (Rodán et al., 2016) and 169 children in grades 4 and 5 (Barel and Tzischinsky, 2018). Another study revealed that boys outperformed girls in a mental rotation task in grades 2–4, whereas in grades 5 and 6 boys and girls performed equally (van Tetering et al., 2019). Furthermore, no sex differences were reported for cognitive flexibility and processing speed in a cohort of Spanish-speaking children aged 9–14 (Arango-Lasprilla et al., 2017).

Given the growing global socioeconomic inequality (Alvaredo et al., 2018), it is essential to consider socioeconomic variables in cognitive research. Previous studies suggested that socioeconomic disparities affect children’s health and development, for example via the associations between higher socioeconomic status and healthier nutrition, less excessive television use, fewer behavioral difficulties, higher quality of life, fewer critical life events, and more physical activity in 3 to 18-year-olds (Poulain et al., 2019), which in turn could shape the cognitive development. Accordingly, children from lower socioeconomic backgrounds tended to experience developmental delays more frequently (Skopek and Passaretta, 2021). Since maternal education is one of the factors determining a family’s socioeconomic status, it was used in studies to describe the children’s sociodemographic environment (Desai and Alva, 1998; Assari, 2020). After controlling for socioeconomic variables, such as family income, maternal education remained a significant determinant of the children’s health and development (Chen and Li, 2009). More educated mothers were more likely to invest in their children by providing books, musical instruments, or special lessons (Carneiro et al., 2013). Accordingly, higher parental education levels were associated with better visual–spatial working memory performance in children (Assari, 2020). Regional different results were observed regarding the association between parental education and children’s cognitive flexibility and processing speed, making it difficult to draw a general conclusion (Arango-Lasprilla et al., 2017). Furthermore, most studies focused on a single cognitive domain within one particular cohort. Consequently, there are still research gaps concerning socioeconomic variables in cognitive research.

There is evidence that children’s performance differed between traditional and game-based cognitive assessment tools (Rosas et al., 2015). Moreover, it was reported that 337 children from kindergarten through third grade preferred game-based over traditional cognitive tests (Rosas et al., 2015). Therefore, we included Tetris, one of the most popular computer games, as an additional exploratory test. The game involves stacking various shapes that fall from the top of the screen to form a horizontally closed line that then disappears, measures cognitive flexibility, visual–spatial working memory, inhibition, processing speed, and strategy development (Lindstedt and Gray, 2015). However, only one study explored Tetris in youth regarding its relationship with other cognitive tests. The study found a significant association between more cleared lines in Tetris and better results in spatial tasks, but no significant associations with other cognitive test results (Lau-Zhu et al., 2017). No other factors, such as sex or parental education, were considered in this survey.

Based on these findings, we hypothesized that children whose mothers had higher education perform better in all tests. Moreover, we expected boys to excel in visual–spatial tasks and girls to perform better in the attention test. We anticipated no significant sex differences regarding cognitive flexibility and processing speed. We hypothesized a significant association between more cleared lines in Tetris and better results in the mental rotation task.

## 2 Methods

### 2.1 LIFE Child study

The data were collected between October 2019 and December 2023 within the LIFE Child study as part of the Leipzig Research Center for Civilization Diseases (LIFE). It is a prospective, longitudinal cohort study conducted in Leipzig (Germany) and focuses on the development of healthy children, but also contains a sub-cohort of children with obesity (Quante et al., 2012; Poulain et al., 2017). The recruitment of participants starts already before birth in the 24th week of pregnancy. The cutoff for including participants in the study is at age 16. The last examination at the research center takes place at age 20. In October 2019, the Continuous Performance Test and, in January 2020, Tetris, the Mental Rotation, and Trail Making Test were included in the study program. Annual visits occur at the research center to gather comprehensive information about each child’s development. The study was conducted in conformity with the Declaration of Helsinki (World Medical Association, 2013). The study protocol was approved by the Ethics Committee of the Medical Faculty of Leipzig University (Reg. No. 477/19-ek). Written informed consent was obtained from legal guardians and children over the age of 12. This research was conducted during the COVID-19 pandemic, a period marked by varying and frequently changing regulations regarding lockdowns in Saxony. Except for March and December 2020, children generally attended school in person, although their leisure activities were restricted for most of the time. The research center was closed from November 2020 to January 2021, during which data collection was limited to online questionnaires. Apart from that, cognitive tests were conducted at the research center as usual, in accordance with hygiene regulations.

### 2.2 Procedure

The day in the research center started around 8 a.m. with an anamnesis interview and blood collection. Since the children had remained fasting for the blood draw, they had a break to eat a meal at the breakfast buffet afterward. During the day they had an individual schedule for all appointments, such as the dentist, sports test, anthropometry, questionnaires, or cognitive tests. To give children a break between the cognitive tests, they were applied in two separate blocks. One block included Tetris, Mental Rotation, and Trail Making Test, and the other block consisted of the Continuous Performance Test. Usually around 1–2 p.m., all scheduled appointments were finished and the visit ended with an appraisal session with one of the study assistants.

### 2.3 Participant selection

**Figure 1:**
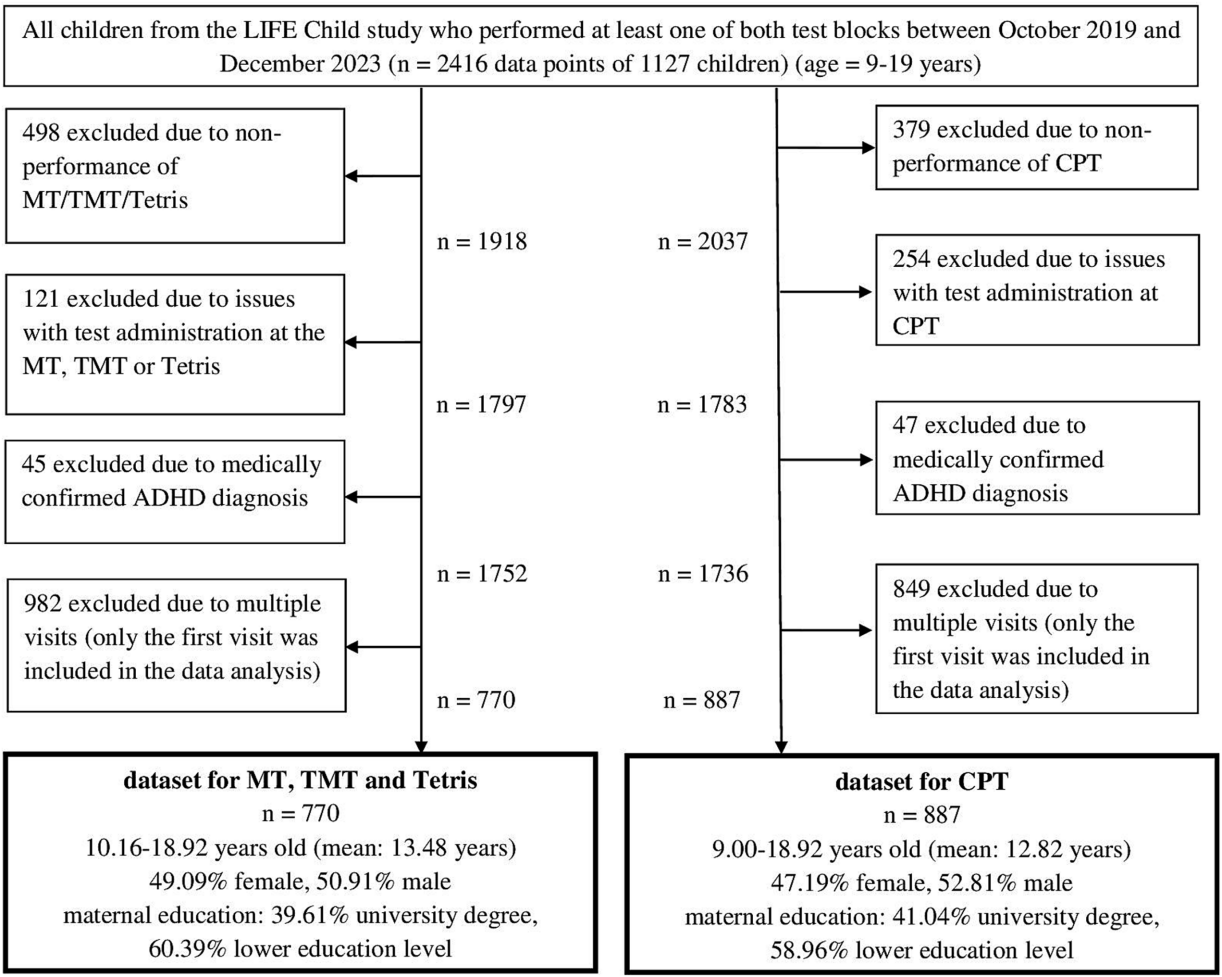
Flowchart of participant selection, CPT, Continuous Performance Test; MT, Mental Rotation Test; TMT, Trail Making Test part B; ADHD, Attention Deficit Hyperactivity Disorder.

Since the test block including Tetris, Mental Rotation, and the Trail Making Test, proved too demanding for younger children, these tasks were administered only from the age of 9 onwards. The Continuous Performance Test was already applied in younger children (age 3.5 and older). However, in this survey we only analyzed data of children aged 9 or older. Data of younger children are published elsewhere (Poulain et al., 2025). All children who performed at least one of both test blocks in the study period 2019–2023 were eligible for analysis (*n* = 2,416 data points of 1,127 children). Consequently, two datasets were created, based on the testing structure. In the case of technical problems or fatigue and distraction of the child, test results could not be analyzed. Finally, in the case of multiple visits per child, only the first test administration was considered, as we observed a significant training effect with increasing visits. Therefore, the final samples comprised 770 children having performed Tetris, Mental Rotation, and Trail Making Test, and 887 children having performed the Continuous Performance Test. 711 participants performed both test blocks. The participant selection process is outlined in Figure 1.

### 2.4 Cognitive tests

Each child received standardized instructions from trained study assistants, and every test began with a short trial run to familiarize participants with the test. During the test administration, the experimenter only observed the proceedings and intervened as little as possible. If an intervention was required due to task misunderstandings or distraction, the study assistant documented it in the final test evaluation for quality control purposes, and the corresponding trial was excluded. All tests have already been used in previous studies with children and adolescents (Reitan, 1971; Conners et al., 2003; Lau-Zhu et al., 2017; Assari, 2020). The software used to create and administer all tasks was developed in-house with JavaScript, based on the protocols of previous studies (Valiente et al., 2010; Alaeddin et al., 2024).

#### 2.4.1 Continuous Performance Test

The Continuous Performance Test assesses attention and inhibition processes (Munkvold et al., 2014; Shaked et al., 2020) and was initially designed for the detection of brain damage (Rosvold et al., 1956). Children and adolescents were seated in front of a screen, where they were shown a series of 210 images [fish, plane, rabbit, ball, boat, chair, umbrella, bricks, kite (Valiente et al., 2010)] sequentially for 7 min. Each picture was shown for 1.5 s with an interstimulus interval of 0.5 s. Participants were instructed to press a green-highlighted key whenever a fish appeared after an airplane. Overall, there were 42 targets. The number of errors of commission (the child reacted although the target was not shown) and omission (the child did not react although the target was shown), along with the mean reaction time for correct clicks were recorded for each child. Fewer errors of omission indicated better selective attention, and fewer errors of commission showed better inhibition control. Shorter reaction times indicated faster processing speed (Munkvold et al., 2014).

#### 2.4.2 Mental Rotation/Little Man Test

The Little Man Test required participants to mentally rotate two-dimensional figures in their visual short-term memory (Luciana et al., 2018). Participants were shown a figure on the screen, holding a bag in either the right or left hand. The figure could be shown in frontal or rear view, upright or upside down. Children used two buttons to decide whether the bag was in the right or left hand. The number of different figure orientations (upright or upside down, frontal or rear view) and bag positions (right or left hand) were equal. The total time for the 32 tasks and the number of errors were measured. Lower total time and fewer errors indicated better cognitive flexibility and visual–spatial working memory (Luciana et al., 2018).

#### 2.4.3 Trail Making Test

In the Trail Making Test part B, participants had to connect numbers (1–12) with letters (A–L) on a screen with the help of a computer mouse as quickly as possible, alternating between the two in correct ascending numerical or alphabetical order. The position of the labels was modeled after the Trail Making Test part B in the Rhineland study (Alaeddin et al., 2024). We analyzed the total time taken, with less time reflecting better mental flexibility, inhibition, and faster processing speed (Espy and Cwik, 2004; Wagner et al., 2011).

#### 2.4.4 Tetris

Tetris involved stacking various shapes that fell from the top of the screen to form a horizontally closed line, which then disappeared. The next shape appeared in the preview window. It was modeled after the original Tetris game but ended automatically after 7 min. We considered the number of cleared lines, reflecting spatial abilities (Lau-Zhu et al., 2017), and the mean number of unnecessary rotations and movements. Fewer rotation and movement faults indicated less impulsivity and better cognitive flexibility. Furthermore, Tetris challenged players to make quick decisions, testing their perception and processing of information, executive functions as inhibition, and strategy development (Lindstedt and Gray, 2015). Since many children already played Tetris before, we assessed and adjusted for their Tetris experience using four categories: 1—never played Tetris before, 2—occasionally played Tetris before, 3—in the past used to play Tetris a lot, and 4—intense Tetris play in the last 6 months.

### 2.5 Maternal education

To characterize the children’s socioeconomic environment, maternal education was captured based on a parental questionnaire. Mothers completed two questions on their highest school degree and their highest professional degree. This information was combined to a score ranging from 1 to 7 (Lampert et al., 2014), with higher scores indicating higher education. For further analysis, we distinguished two groups of children: those whose mothers had the highest score (7), indicating a university degree, and those with lower scores (1–6), indicating lower education.

### 2.6 Data analysis

R version 4.3.1 was used for all statistical computations and visualizations. We used multiple linear regression models, incorporating the number of errors of omission/commission and mean reaction time for a correct click for the Continuous Performance Test, the sum of errors and required time for the Mental Rotation Test, the number of completed lines and mean rotation/movement faults in Tetris, and the total time for the Trail Making Test as dependent variables. Sex and highest maternal education were included as categorical independent variables. All models were adjusted for age as a continuous numeric variable, while the Tetris models were further adjusted for the Tetris experience. Interactions between age, sex, and maternal education were investigated but only reported if they were statistically significant (*p* < 0.05). Since boys were expected to develop later and girls to peak earlier, we initially used a polynomial model with respect to age concerning the Trail Making Test. However, the parameters for the 2nd and 3rd degrees were insignificant, so we opted for a linear model at this point. To generate a composite score from the three standard tests and compare it with Tetris, we calculated *z*-scores for all four tests, adjusted for age, sex, and, in the case of Tetris, for Tetris experience, using the gamlss package (Rigby and Stasinopoulos, 2005). The Box-Cox-Cole-Green (BCCG) distribution was applied to model the Trail Making Test, and Zero Inflated Poisson (ZIP) to model cleared lines in Tetris and errors in the Mental Rotation and Continuous Performance Test. Strengths of associations were indicated by non-standardized regression coefficients (*ß*). The significance level was *α* = 0.05. Plots, created with ggplot, included grey shadows to show the 95%-confidence interval (95% CI). An ordinal regression analysis examined the associations between Tetris experience (dependent variable), sex, and maternal education. These results were reported as odds ratios (OR).

## 3 Results

All results, including mean values, standard deviations, minimums, and maximums, are summarized in Tables 1, 2. Non-standardized regression coefficients (*ß*) and *p*-values will only be reported for significant associations in the following sections. The supplementary material includes a table (Supplementary Table S1) detailing the main effects of age, sex, and maternal education, their interactions, and the relative *t*-statistics.

**Table 1:** Mean values, standard deviations, minimum, and maximum for each test.

Test	Test variables	*M*	*SD*	Min	Max
Continuous Performance Test	Omission errors	1.53	2.96	0	41
	Commission errors	2.70	4.90	0	60
	Reaction time (ms)	424.47	84.75	146.00	855.17
Mental Rotation Test	Sum error	6.31	5.94	0	25
	Sum time (s)	82.10	37.27	19.49	348.16
Trail Making Test	Sum time (s)	47.25	17.28	17.85	187.89
Tetris	Cleared lines	11.34	8.48	0	44
	Mean rotation faults	1.03	0.47	0	3
	Mean movement faults	1.00	0.52	0	3.41

**Table 2:** Associations between the children’s results in four cognitive tests, sex, age, and maternal education.

Cognitive tests	Test variables	Independent variables					
		Sex: male^a^		Education mother: the highest education level^b^		Age	
		*ß* (95% CI)	*p*	*ß* (95% CI)	*p*	*ß* (95% CI)	*p*
Continuous Performance Test	Omission errors	**0.79 (0.22 to 1.35)** ^**c**^	**0.007**	**−0.72 (−1.11 to −0.33)**	**<0.001**	−0.03 (−0.14 to 0.08)^c^	0.578
	Commission errors	**1.89 (0.96 to 2.82)** ^**d**^	**<0.001**	**−1.04 (−1.69 to −0.40)**	**0.002**	−0.14 (−0.32 to 0.04)^d^	0.140
	Reaction time (ms)	0.12 (−10.29 to 10.53)	0.982	3.04 (−7.61 to 13.69)	0.576	**−12.03 (−14.06 to −10.01)**	**<0.001**
Mental Rotation Test	Sum error	−0.21 (−1.01 to 0.58)	0.598	**−1.24 (−2.06 to −0.42)**	**0.003**	**−0.85 (−1.02 to −0.68)**	**<0.001**
	Sum time (s)	−1.04 (−6.21 to 4.02)	0.674	3.59 (−1.67 to 8.85)	0.181	**−4.00 (−5.10 to −2.89)**	**<0.001**
Trail Making Test	Sum time (s)	1.95 (−0.28 to 4.17)	0.086	−0.48 (−2.77 to 1.81)	0.681	**−3.14 (−3.62 to −2.66)**	**<0.001**
Tetris	Cleared lines	**2.46 (1.47 to 3.45)**	**<0.001**	0.96 (−0.04 to 1.95)	0.059	**1.56 (1.34 to 1.77)**	**<0.001**
	Mean rotation faults	**0.10 (0.04 to 0.17)**	**0.003**	0.00 (−0.07 to 0.07)	0.989	0.00 (−0.02 to 0.01)	0.962
	Mean movement faults	**0.30 (0.23 to 0.37)**	**<0.001**	−0.06 (−0.13 to 0.02)	0.125	−0.01 (−0.02 to 0.01)	0.306

a Reference = female.

b Reference = lower education.

c Significant interaction between age and sex, decreases 0.23 per additional year for boys (*p* = 0.002).

d Significant interaction between age and sex, decreases 0.37 per additional year for boys (*p* = 0.003).

Significant associations are highlighted in bold (CI = confidence interval).

### 3.1 Participant characteristics

The first dataset, including Tetris, Mental Rotation, and Trail Making Test, contained 770 participants (49.09% girls, 50.91% boys) aged between 10 and 18 years (mean age 13.84). 305 mothers (39.61%) had the highest education. For the Continuous Performance Test, 887 adolescents were included (47.19% girls, 52.81% boys), aged 9 to 18 (mean age 12.82). In this group, 364 mothers (41.04%) had the highest education.

### 3.2 Continuous Performance Test

**Figure 2:**
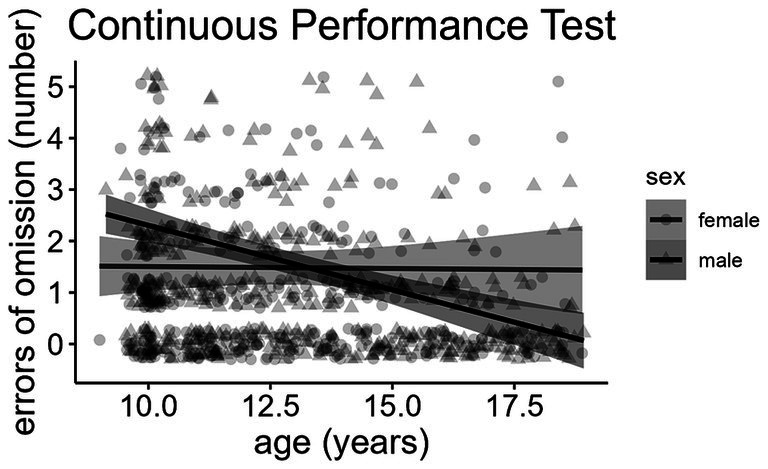
Age-dependent sex differences regarding the number of errors of omission in the Continuous Performance Test.

We observed a significant age-dependent sex interaction regarding the number of omission (Figure 2) and commission errors. Boys made more omission errors (*ß* = 0.79 omission errors, *p* = 0.007) and commission errors (*ß* = 1.89 commission errors, *p* < 0.001) than girls at the age of 10 years. Still, they improved with age (*ß* = −0.23 omission errors/year, *p* = 0.002; *ß* = −0.37 commission errors/year, *p* = 0.003) and ultimately made fewer omission and commission errors than girls, with small effect sizes (girls made 1.37 more omission errors and 1.56 more commission errors than boys at age 18.92). Conversely, girls maintained an almost constant performance trajectory over the entire age range. There was no significant association between sex and the mean reaction time. High maternal education was associated with significantly fewer errors of omission (*ß* = −0.72 omission errors, *p* < 0.001) and commission (*ß* = −1.04 commission errors, *p* = 0.002) in the children’s Continuous Performance Test. However, there was no significant association between maternal education and the children’s reaction time. Older participants had significantly faster reaction times (*ß* = −12.02 milliseconds/year, *p* < 0.001) than younger children.

### 3.3 Mental Rotation/Little Man Test

**Figure 3:**
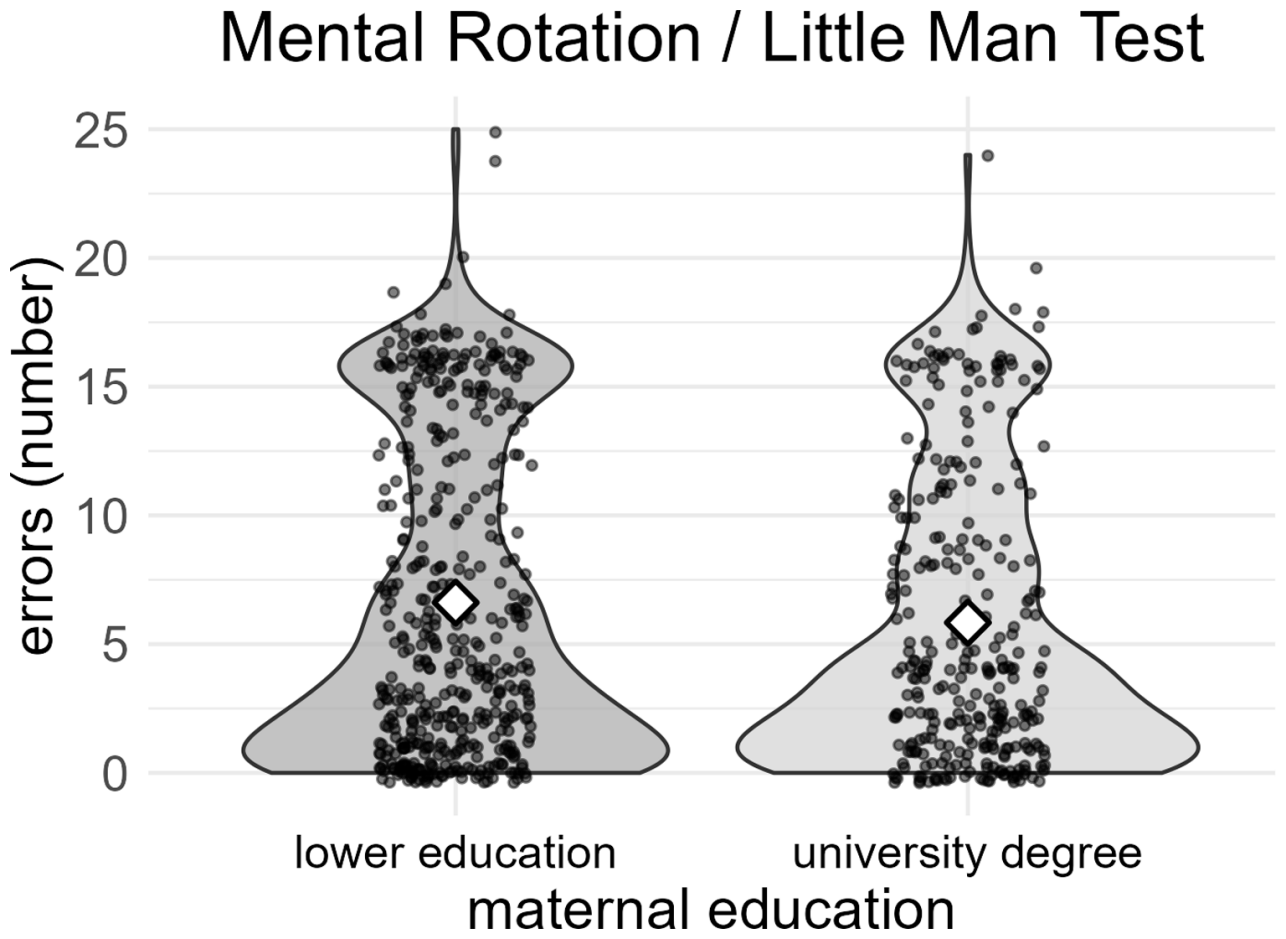
Differences in the number of errors in the Mental Rotation Test based on maternal education.

No significant sex differences were observed regarding the number of errors and the total time in the Little Man Test. Children whose mothers had the highest education made significantly fewer errors (*ß* = −1.24 errors, *p* = 0.003) (Figure 3). The association between maternal education and total time in the Little Man Test was not significant. Older participants made significantly fewer errors (*ß* = −0.85 errors/year, *p* < 0.001) and needed less time (*ß* = −4.00 s/year, *p* < 0.001) than younger children.

### 3.4 Trail Making Test

There were no significant associations between sex or maternal education level and the total time taken in the Trail Making Test. Older participants were significantly faster (*ß* = −3.14 s/year, *p* < 0.001) than younger children.

### 3.5 Tetris

**Figure 4:**
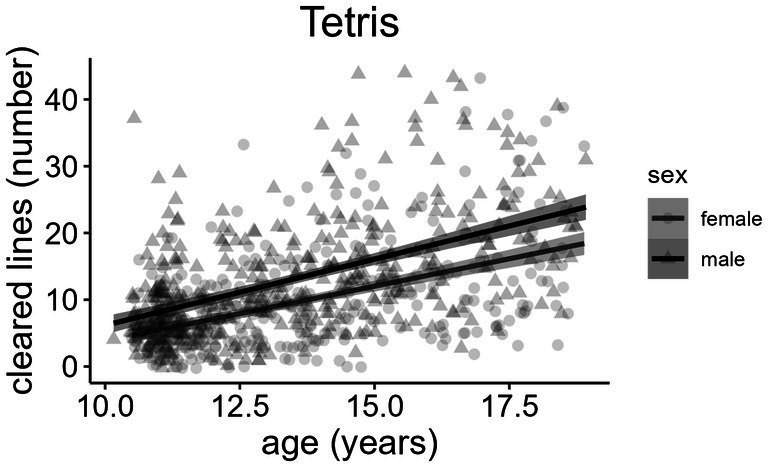
Sex differences in the number of cleared lines in Tetris.

Boys had significantly higher Tetris experience levels compared to girls [OR = 2.53, 95% CI (1.80, 3.60), *p* < 0.001]. Even after adjusting for Tetris experience, boys achieved significantly more cleared lines than girls across all ages (*ß* = 2.46 lines, *p* < 0.001) (Figure 4). However, boys also made significantly more movement (*ß* = 0.30 movement faults, *p* < 0.001) and rotation faults (*ß* = 0.10 rotation faults, *p* = 0.003) than girls. The association between maternal education and Tetris experience was not significant [OR = 0.96, 95% CI (0.91, 1.02), *p* = 0.189], and no significant differences in children’s Tetris performance based on their mother’s education level were observed. Older participants achieved significantly more cleared lines (*ß* = 1.56 lines/year, *p* < 0.001) than younger children. The *z*-score of Tetris performance was significantly associated with the total time in the Trail Making Test (*ß* = 0.32, *p* < 0.001) and the errors in the Mental Rotation Test (*ß* = 0.13, *p* = 0.002). No significant association was observed between Tetris performance and omission or commission errors in the Continuous Performance Test (*ß* = 0.07, *p* = 0.085). The model explained 6.8% of the variance in Tetris performance (*R*^2^ = 0.07).

## 4 Discussion

Cognitive abilities develop through an active process, which is shaped by interactions with the environment (Karmiloff-Smith, 1994). Important environmental factors might be sex and parental education. This study examined the associations between sex, maternal education, and cognitive performance using three standard tests in a large cohort of healthy children and adolescents, aged 9–19 years. Since children might prefer game-based cognitive tests over traditional assessment methods (Rosas et al., 2015), we further investigated Tetris as an additional exploratory test in childhood and adolescence more detailed for the first time. Significant sex differences were found in the Continuous Performance Test and Tetris, while maternal education was associated with differences in the Continuous Performance and Mental Rotation Test results. Although all tests assessed multiple cognitive domains with some overlap, such as cognitive flexibility, attention, and processing speed, the extent to which each cognitive domain contributed to the performance varied across tests, leading to differences in the results.

### 4.1 Age

Older participants performed significantly better across all tests, except for the number of mean rotation and movement faults in Tetris, which aligns with previous studies (Reitan, 1971; Quaiser-Pohl et al., 2006). Since no prior studies examined Tetris in childhood regarding age, we could only suggest that the number of cleared lines more directly reflected cognitive abilities such as visual–spatial abilities and processing speed, which tend to improve with age. In contrast, rotation and movement faults might be influenced more by individual playing style, motor control, or prior gaming experience, resulting in greater variability independent of age.

### 4.2 Sex differences

Previous studies concerning sex differences in cognitive tests yielded mixed results (Quaiser-Pohl et al., 2006; Rodán et al., 2016; Barel and Tzischinsky, 2018; van Tetering et al., 2019). In this study, younger boys were less attentive and more impulsive than girls in the Continuous Performance Test until age 13.29 for omission errors and 14.91 for commission errors, but improved during puberty and eventually surpassed girls in attention control. In contrast, girls maintained a stable performance over the entire age range. Interestingly, no sex differences were found regarding processing speed, measured by the reaction time. Notably, these patterns were not reported in any of the mentioned literature. For example, Conners et al. (2003) found that participants improved their Continuous Performance Test performance with age, but boys made more errors and had faster reaction times over the entire age span (9–17 years), which was explained by the boys’ tendency to respond impulsively and take more risks than girls. Differences in test duration and setting might explain these discrepancies. Conners et al. administered a 14-min test version at participants’ homes, whereas the LIFE Child study employed a 7-min version in a distraction-free research room. Consequently, we cannot infer how children would have performed over a longer duration in their home with more external stimuli. The discrepancy in reaction time findings could be explained by changes in computer usage over time. When Conners et al. (2003) published their study, boys were generally more familiar and self-confident in the use of computers than girls (Colley and Comber, 2003), contributing to faster reaction times. With the increasing integration of computers in schools and the rise of gaming among girls (Dixon and Boudreau, 2009; OECD, 2020), the differences in reaction time might have diminished. Further research could investigate whether training attention and inhibition control in young boys reduces sex differences in young children and supports overall development.

Even after adjusting for Tetris experience, boys cleared more lines than girls but made more rotation and movement faults. We conclude that boys were more courageous and dared to rotate and move the figures more than girls, resulting in more cleared lines. This aligns with the assumption that boys take greater risks in cognitive tests (Conners et al., 2003; Andreoni et al., 2020). Additionally, the observed differences may be attributed to boys’ greater experience with video games, leading to faster reactions and more confident exploratory behavior. Although gaming has become more common among girls (Dixon and Boudreau, 2009), boys still play video games, especially action games, more frequently, whereas girls are more likely to use computers for social networking (Fairlie, 2016). This explanation is supported by the assumption that Tetris most closely resembles video games, such as action games, compared to other cognitive tests, requiring quick decision-making and fast reactions (Lindstedt and Gray, 2015). As our study did not include video gaming experience as a further covariate, we recommend it for future research.

Contrary to our hypothesis and previous studies (Quaiser-Pohl et al., 2006), no significant sex differences were observed in the Mental Rotation Test. Nevertheless, our results align with the study of Barel and Tzischinsky (2018), who found no sex differences in 169 children in grades 4 and 5 in a 2D-computerized test and van Tetering et al. (2019), who found no sex differences in a mental rotation task in grades 5 and 6. Since there was a positive correlation between the number of mathematics courses taken and better performance in visual–spatial tasks (Burnett and Lane, 1980), this change may be attributed to equal educational opportunities for both sexes. However, our findings could not be generalized to all societies, as cultural differences concerning gender equity in school enrollment were reported (Else-Quest et al., 2010). Furthermore, male-typical activities, such as playing with construction sets or action video games, could improve spatial abilities (Miller and Halpern, 2013; Uttal et al., 2013). Cultural shifts toward gender-neutral play provide both sexes equal opportunities to playfully train their visual–spatial working memory from an early age.

No significant sex differences in cognitive flexibility and processing speed were found based on the results in the Trail Making Test and the reaction time in the Continuous Performance Test. These findings align with previous studies in smaller cohorts (Reitan, 1971; Leòn-Carriòn, 1989) and with normative data for the Trail Making Test in Spanish-speaking pediatric populations (Arango-Lasprilla et al., 2017), suggesting that these cognitive abilities were shaped by individual differences in the brain structure, such as the cerebellum-frontal network (Wong et al., 2021), rather than sex differences.

### 4.3 Maternal education

Along with sex, maternal education seemed to play a considerable role in evaluating cognitive test results, although its effects varied across tests. Children whose mothers had higher education levels were more attentive and less impulsive in the Continuous Performance Test, which may be related to its 7-min duration and monotonous procedure requiring more self-control to sustain prolonged concentration. Self-control is defined as the ability to resist automatic impulses and deliberately regulate one’s behavior (Bin et al., 2013). Since lower socioeconomic status was associated with less self-control (Ng-Knight and Schoon, 2017), children whose mothers had lower education levels might become distracted more quickly during the test. Children from higher-educated families may have been able to sustain their attention span and inhibition control longer due to better self-control skills. This hypothesis was additionally supported by the results in the Trail Making Test. Since it has a more stimulating character, shorter duration, and the total time depends on the child (minimal duration: 17.80 s, maximal duration: 3.13 min, mean: 47.25 s), all participants seemed to remain concentrated, as no significant differences were found based on maternal education. Furthermore, cognitive processing speed, measured among other cognitive domains in the Trail Making Test, had been linked to a cerebellum-frontal network (Wong et al., 2021) rather than being shaped by social environmental factors, such as education or financial resources. This might explain the absence of significant associations between maternal education and both Trail Making Test performance and reaction time in the Continuous Performance Test, reflecting processing speed. Since normative data for Spanish-speaking populations found regional differences concerning the association between parental education and Trail Making Test results (Arango-Lasprilla et al., 2017), we cannot extrapolate our findings to other countries. In this context, lower maternal education might be considered as a risk factor for poorer cognitive abilities and less favorable developmental outcomes in adulthood, as problems in sustained attention in childhood were linked to health problems (Kubzansky et al., 2009), socioeconomic disadvantages (Galéra et al., 2012), cigarette smoking, and delinquent behavior (Casseus et al., 2025). Therefore, early, targeted support should be provided to the affected children.

Although the Little Man Test has a shorter duration than the Continuous Performance Test and the total time depends on the child (minimal duration: 19.49 s, maximal duration: 5.80 min, mean: 82.10 s), higher maternal education was associated with better mental rotation abilities, similar to the results of the ABCD study (Assari, 2020). A possible explanation could be that on average children took almost twice as long to complete the Little Man Test compared to the Trail Making Test. Additionally, the Little Man Test might not be as stimulating as the Trail Making Test. Maintaining concentration during the Mental Rotation task might demand greater self-control than the Trail Making Test. Moreover, higher maternal education was linked to increased family income (Maryana et al., 2024). Accordingly, more educated mothers had more financial resources to provide books, musical instruments, special lessons, and the availability of a computer or construction toys to train the children’s visual–spatial abilities (Carneiro et al., 2013). Providing targeted support to train the visual–spatial working memory in kindergarten and school could help mitigate social disparities, as previous studies emphasized its importance for education (Liu et al., 2021), particularly in mathematics, science, technology, and engineering fields (Verdine et al., 2017; Cui and Guo, 2022). When examining the association between maternal education and performance in the Mental Rotation Test in Figure 3, it is conspicuous that a substantial number of participants made exactly 16 errors. These 73 participants did not appear to apply any mental rotation strategy and consistently selected the side of the screen where the bag was located, regardless of the stimulus orientation.

Interestingly, no differences based on maternal education emerged in Tetris, despite its 7-min duration. Children from less educated families might prefer game-based tests over traditional tests, partly due to anxiety triggered by traditional tests (Rosas et al., 2015). Therefore, their potential might be better tested in gaming-like tests such as Tetris.

### 4.4 Associations with Tetris performance

Tetris performance was significantly associated with the Trail Making and Mental Rotation Test, but not with the Continuous Performance Test, aligning with a previous study linking mental rotation and Tetris performance in adolescents (Lau-Zhu et al., 2017). The association with the Trail Making Test, measuring cognitive flexibility and processing speed (Reitan, 1971), reflects that Tetris engaged multiple cognitive domains (Lindstedt and Gray, 2015). Both the Continuous Performance Test and Tetris required sustained performance over 7 min. Unlike the monotonous and feedback-free Continuous Performance Test, Tetris is more dynamic and provides continuous feedback, which might enhance sustained attention. The regression model explained 6.8% of the variance in Tetris performance, suggesting that the three standard tests captured only a small part of the cognitive processes involved in Tetris and that each test targeted different cognitive domains. Therefore, using multiple cognitive assessments might be essential to obtain a comprehensive understanding of children’s cognitive functioning.

### 4.5 Strength and limitations

Since we studied a cohort with an above-average socioeconomic status (Poulain et al., 2017), it may not represent the entire population. Moreover, only maternal education, categorized dichotomously, was used as a measure of socioeconomic status. Excluding multiple visits led to an uneven age distribution, with fewer older participants. Additionally, we observed a ceiling effect in the Continuous Performance and Mental Rotation Test, making further differentiation between the participants difficult. Since the study was conducted during COVID-19, potential effects of the pandemic on attention performance cannot be excluded. The study’s strengths include the large sample size, wide age range, and simultaneous examination of four cognitive tests in a cohort of healthy children. Furthermore, we investigated Tetris as a cognitive test in childhood and adolescence more detailed for the first time.

### 4.6 Conclusion

Age and maternal education affected the cognitive performance of boys and girls differently. This knowledge impacts how cognitive performance is evaluated in school or psychological assessments. Distinguishing between physiological variety, such as the delayed improvement of boys in the Continuous Performance Test, and cognitive delay is essential to know when and how individual support should be provided. Lower maternal education emerged as a potential risk factor for poorer cognitive abilities. We recommend future longitudinal studies to examine whether targeted interventions not only enhance cognitive skills but also lead to long-term improvements in these developmental outcomes. Moreover, we recommend further investigating Tetris as an alternative game-based assessment tool for children and adolescents.

## Data Availability

The raw data supporting the conclusions of this article is not readily available as access requests are managed by the LIFE Child data use and access committee. To request access, please reach out to life-dm@lists.uni-leipzig.de. Further inquiries can be directed to the corresponding author.
