# Supplementary material for: Sociodemographic correlates of cognitive performance in healthy children and adolescents
Source: Front Psychol. 2025 Nov 20;16:1656310. doi: 10.3389/fpsyg.2025.1656310 (PMC12678859; doi:10.3389/fpsyg.2025.1656310)
Supplement: Supplementary file 1 [file Table_1.docx]

**Table S1:** The main effects of age, sex, and maternal education, their interactions, and the relative t-statistics.

| **test** |  |  | **ß (95% CI)** | **t** | ***p*** |
| --- | --- | --- | --- | --- | --- |
| **Continuous Performance Test** | **omission errors** | **age** | - 0.03 (- 0.14 to 0.08) | - 0.56 | .578 |
|  |  | **sex: male ^a^** | **0.79 (0.22 to 1.35)** | **2.72** | **.007** |
|  |  | **maternal education: the highest education level ^b^** | **- 0.72 (- 1.11 to - 0.33)** | **- 3.60** | **<.001** |
|  |  | **age*sex** | **- 0.23 (- 0.38 to - 0.08)** | **- 3.06** | **.002** |
|  |  | **age*maternal education** | - 0.02 (- 0.18 to 0.13) | - 0.28 | .779 |
|  |  | **sex*maternal education** | 0.47 (- 0.32 to 1.26) | 1.17 | .241 |
|  |  | **age*sex*maternal education** | 0.26 (- 0.04 to 0.57) | 1.68 | .093 |
|  | **commission errors** | **age** | - 0.14 (- 0.32 to 0.04) | - 1.48 | .140 |
|  |  | **sex: male ^a^** | **1.89 (0.96 to 2.82)** | **3.99** | **<.001** |
|  |  | **maternal education: the highest education level ^b^** | **- 1.04 (- 1.69 to - 0.40)** | **-3.17** | **.002** |
|  |  | **age*sex** | **- 0.37 (- 0.62 to - 0.13)** | **- 3.00** | **.003** |
|  |  | **age*maternal education** | 0.15 (- 0.11 to 0.40) | 1.13 | .256 |
|  |  | **sex*maternal education** | 0.81 (- 0.49 to 2.10) | 1.22 | .222 |
|  |  | **age*sex*maternal education** | 0.16 (- 0.35 to 0.66) | 0.60 | .546 |
|  | **reaction time (ms)** | **age** | **-12.03 (- 14.06 to - 10.01)** | **- 11.67** | **<.001** |
|  |  | **sex: male ^a^** | 0.12 (- 10.30 to 10.51) | 0.02 | .982 |
|  |  | **maternal education: the highest education level ^b^** | 3.04 (- 7.61 to 13.69) | 0.56 | .576 |
|  |  | **age*sex** | 0.08 (- 3.99 to 4.15) | 0.04 | .970 |
|  |  | **age*maternal education** | - 2.35 (- 6.51 to 1.81) | - 1.11 | .268 |
|  |  | **sex*maternal education** | 10.62 (- 10.78 to 32.01) | 0.97 | .330 |
|  |  | **age*sex*maternal education** | - 0.85 (- 9.23 to 7.53) | - 0.20 | .843 |
| **Mental Rotation Test** | **sum error** | **age** | **- 0.85 (- 1.02 to - 0.68)** | **- 9.69** | **<.001** |
|  |  | **sex: male ^a^** | - 0.21 (- 1.01 to 0.58) | - 0.53 | .598 |
|  |  | **maternal education: the highest education level ^b^** | **- 1.24 (- 2.06 to - 0.42)** | **- 2.98** | **.003** |
|  |  | **age*sex** | - 0.15 (- 0.49 to 0.20) | - 0.84 | .403 |
|  |  | **age*maternal education** | - 0.03 (- 0.38 to 0.33) | - 0.15 | .884 |
|  |  | **sex*maternal education** | - 1.10 (- 2.74 to 0.54) | - 1.32 | .188 |
|  |  | **age*sex*maternal education** | 0.32 (- 0.39 to 1.03) | 0.88 | .378 |
|  | **sum time (s)** | **age** | **- 4.00 (- 5.10 to - 2.89)** | **- 7.10** | **<.001** |
|  |  | **sex: male ^a^** | - 1.04 (- 6.21 to 4.02) | -0.42 | .674 |
|  |  | **maternal education: the highest education level ^b^** | 3.59 (- 1.67 to 8.85) | 1.34 | .181 |
|  |  | **age*sex** | - 0.73 ( - 2.95 to 1.48) | - 0.65 | .517 |
|  |  | **age*maternal education** | 0.28 (- 2.00 to 2.56) | 0.24 | .810 |
|  |  | **sex*maternal education** | 0.48 ( -10.08 to 11.03) | 0.09 | .930 |
|  |  | **age*sex*maternal education** | 0.53 (- 4.05 to 5.10) | 0.23 | .821 |
| **Trail Making Test** | **sum time (s)** | **age** | **- 3.14 (- 3.62 to -2.66)** | **- 12.84** | **<.001** |
|  |  | **sex: male ^a^** | 1.95 (- 0.28 to 4.17) | 1.72 | .086 |
|  |  | **maternal education: the highest education level ^b^** | - 0.48 (- 2.77 to 1.81) | - 0.41 | .681 |
|  |  | **age*sex** | - 0.55 (- 1.52 to 0.41) | - 1.13 | .259 |
|  |  | **age*maternal education** | - 0.49 (- 1.48 to 0.50) | - 0.98 | .330 |
|  |  | **sex*maternal education** | 1.76 (- 2.82 to 6.34) | 0.76 | .450 |
|  |  | **age*sex*maternal education** | 1.24 (- 0.74 to 3.23) | 1.23 | .219 |
| **Tetris** | **cleared lines** | **age** | **1.56 (1.34 to 1.77)** | **14.13** | **<.001** |
|  |  | **sex: male ^a^** | **2.46 (1.47 to 3.45)** | **4.88** | **<.001** |
|  |  | **maternal education: the highest education level ^b^** | 0.96 (- 0.04 to 1.95) | 1.89 | .059 |
|  |  | **Tetris experience** | **3.29 (2.57 to 4.00)** | **9.03** | **<.001** |
|  | **rotation faults** | **age** | 0.00 (- 0.02 to 0.01) | - 0.05 | .962 |
|  |  | **sex: male ^a^** | **0.10 (0.04 to 0.17)** | **2.99** | **.003** |
|  |  | **maternal education: the highest education level ^b^** | 0.00 (- 0.07 to 0.07) | - 0.01 | .989 |
|  |  | **Tetris experience** | **- 0.06 (- 0.11 to - 0.01)** | **- 2.55** | **0.011** |
|  | **movement faults** | **age** | - 0.01 (- 0.02 to 0.01) | - 1.02 | .306 |
|  |  | **sex: male ^a^** | **0.30 (0.23 to 0.37)** | **8.11** | **<.001** |
|  |  | **maternal education: the highest education level ^b^** | - 0.06 (- 0.13 to 0.02) | - 1.54 | .125 |
|  |  | **Tetris experience** | - 0.03 (- 0.08 to 0.03) | - 0.97 | .331 |

**^a^ reference = female**

**^b^ reference = lower education**
